# Rapid, Facile Detection of Heterodimer Partners for Target Human G-Protein-Coupled Receptors Using a Modified Split-Ubiquitin Membrane Yeast Two-Hybrid System

**DOI:** 10.1371/journal.pone.0066793

**Published:** 2013-06-21

**Authors:** Yasuyuki Nakamura, Jun Ishii, Akihiko Kondo

**Affiliations:** 1 Department of Chemical Science and Engineering, Graduate School of Engineering, Kobe University, Kobe, Japan; 2 Organization of Advanced Science and Technology, Kobe University, Kobe, Japan; University of Oldenburg, Germany

## Abstract

Potentially immeasurable heterodimer combinations of human G-protein-coupled receptors (GPCRs) result in a great deal of physiological diversity and provide a new opportunity for drug discovery. However, due to the existence of numerous combinations, the sets of GPCR dimers are almost entirely unknown and thus their dominant roles are still poorly understood. Thus, the identification of GPCR dimer pairs has been a major challenge. Here, we established a specialized method to screen potential heterodimer partners of human GPCRs based on the split-ubiquitin membrane yeast two-hybrid system. We demonstrate that the mitogen-activated protein kinase (MAPK) signal-independent method can detect ligand-induced conformational changes and rapidly identify heterodimer partners for target GPCRs. Our data present the abilities to apply for the intermolecular mapping of interactions among GPCRs and to uncover potential GPCR targets for the development of new therapeutic agents.

## Introduction

The potentially large functional and physiological diversity of dimerization among G-protein-coupled receptors (GPCRs) has generated a great deal of excitement due to the opportunity for novel drug discovery [1], [2]. The findings of physiologically relevant GPCR dimers raise the prospect of developing new drugs against a wide range of diseases by focusing on the machinery of targeted dimers because ligand-induced conformational changes in GPCR dimers could affect ligand affinity and signaling function [3], [4]. Since the human genome encodes more than 800 GPCR genes [5], the possible combinations of physiologically significant GPCR heterodimers would be immeasurable. However, due to the existence of numerous combinations, the sets of GPCR dimers are almost entirely unknown and thus their dominant roles are still poorly understood.

Techniques to observe the dimerization of GPCRs include atomic force microscopy, electrophoresis, co-immunoprecipitation, cross-linkage, and fluorescence and bioluminescence resonance energy transfer (FRET and BRET) [3], [4], [6]. The FRET and BRET approaches are especially helpful for in vivo analysis and therefore are widely used for the studies of dimerized GPCRs. However, although the FRET and BRET techniques permit the direct monitoring of GPCR dimerization, it might be difficult to use these techniques to achieve rapid and facile identification of dimerizable candidates among numerous GPCR combinations.

To overcome this limitation, here we established a specialized method to screen candidate heterodimer partners for target GPCRs based on the split-ubiquitin membrane yeast two-hybrid method. In addition, since our system is independent from the activation of mitogen-activated protein kinase (MAPK) signal, it permits not only the identification of heterodimer partners, but also the monitoring of ligand-induced conformational changes.

## Results and Discussion

**Figure 1:**
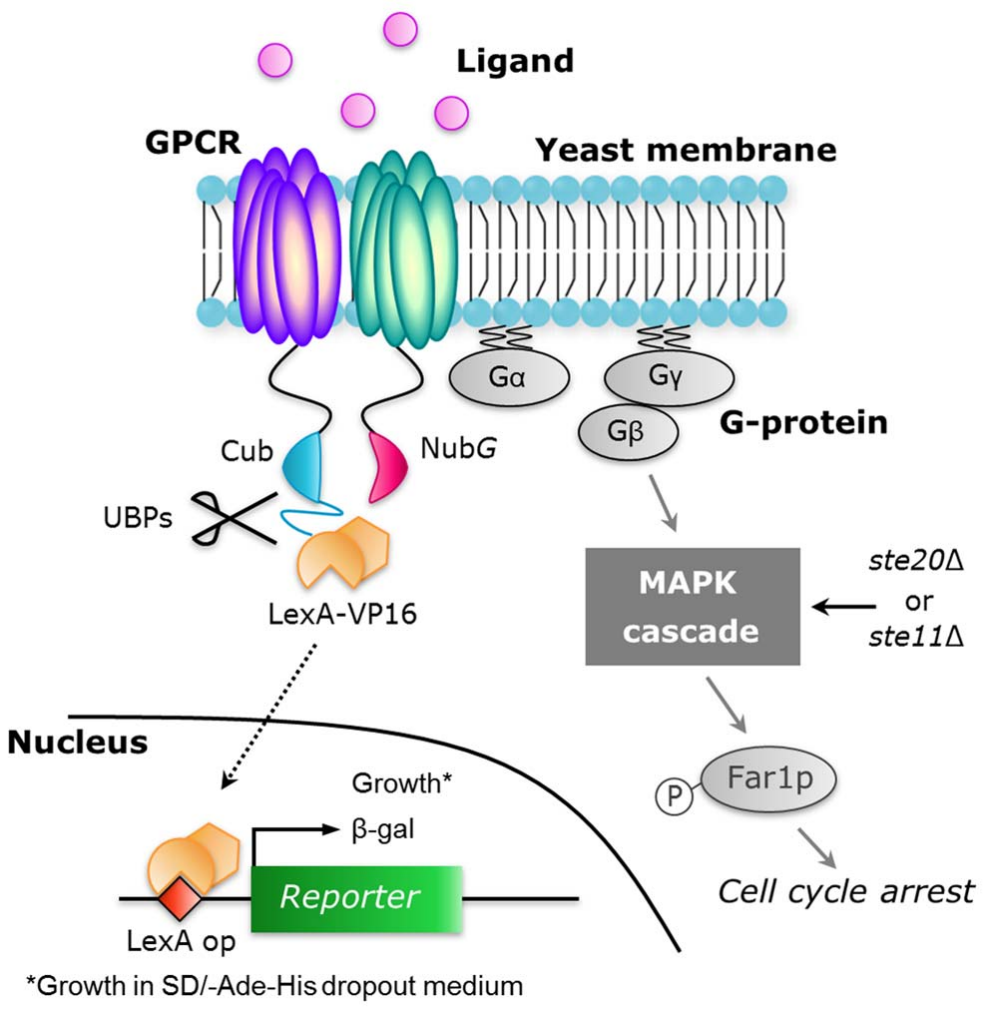
Schematic illustration of yeast pheromone signaling pathway and principle for GPCR dimerization assay based on split-ubiquitin system in yeast. Agonistic ligand binding to the GPCR leads to the activation of heterotrimeric G-proteins, the mitogen-activated protein kinase (MAPK) cascade and a cyclin-dependent kinase inhibitor Far1p. Phosphorylated Far1p induces G1 cell-cycle arrest. The *STE20* or *STE11* gene located upstream of the MAPK cascade was disrupted in the NMY51 strain. In the split-ubiquitin yeast two-hybrid system, Nub*G* will only efficiently interact with Cub when the proteins to which the two split tags are attached interact with each other, resulting in the formation of a Nub*G*/Cub complex. This complex is recognized by ubiquitin-specific proteases (UBPs), which release the artificial transcription factor (LexA-VP16) from the Cub-containing construct. LexA-VP16 then enters the nucleus via diffusion and binds to the LexA-binding sites upstream of the reporter genes. In this study, the GPCRs are fused to the split-ubiquitin and are expressed in MAPK-defective mutant yeast strain of NMY51 to allow the monitoring of GPCR dimerizations and conformational changes responding to binding of ligand.

**Figure 2:**
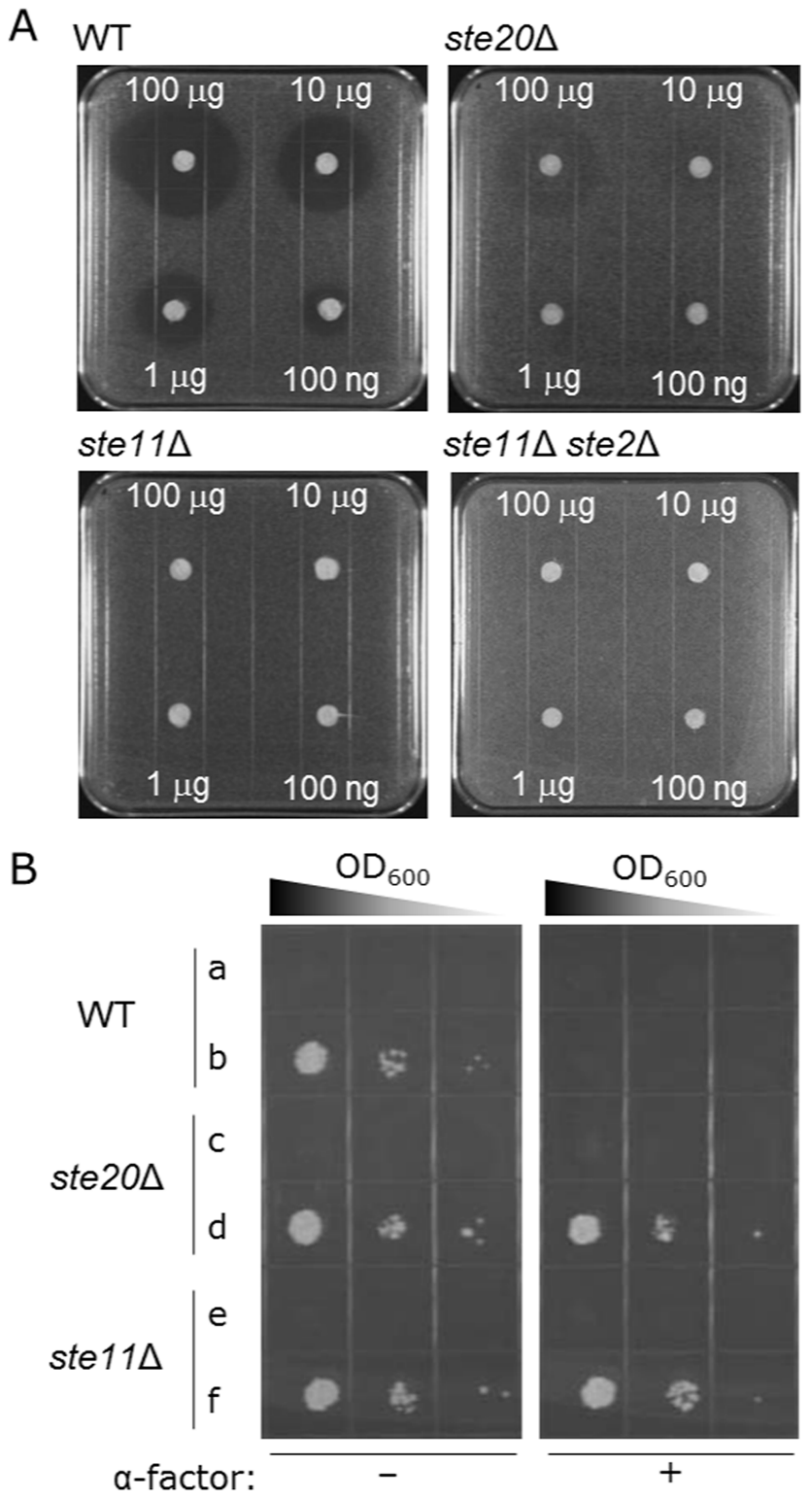
*ste11*Δ allele allowed more strict avoidance of signal-promoted growth arrest in the presence of ligand. (A) Halo-bioassay (agonist-induced growth arrest assay) for *STE20*-, *STE11*- and *STE2*-gene-disrupted strains: NMY51 (WT); NMY61 (*ste20*Δ); NMY62 (*ste11*Δ); and NMY63 (*ste11*Δ *ste2*Δ). Each paper filter disk was spotted with the indicated amount of α-factor. (B) Growth assay of NMY51 (WT; **a,b**), NMY61 (*ste20*Δ; **c,d**) and NMY62 (*ste11*Δ; **e,f**) strains on SD –Leu, Trp, Ade and His dropout plates. Yeast strains harboring pBT3-C/pPR3-C or pCCW-Alg5/pAI-Alg5 respectively expressed Cub/Nub*G* (negative control; **a,c,e**) or Alg5-Cub/Alg5-Nub*I* (positive control; **b,d,f**). Each cell was spotted in serial 10-fold dilutions on selective agar plates with or without 5 µM of α-factor. Nub*I* is a WT Nub tag and interacts spontaneously with Cub.

While the original split-ubiquitin system enables comprehensive screening of protein-protein interactions [7], it is intrinsically possible that the addition of ligand triggers activation of pheromone signaling via endogenous yeast heterotrimeric G-protein [8]. The activation of pheromone signaling in yeast induces cell cycle arrest in G1 phase and triggers global changes in transcription of mating-related genes [9]. The ligand-induced G1 arrest that is exposed as robust growth inhibition in yeast cells [10] might lead to an inadequate assessment of reporter gene activity. Therefore, we constructed a yeast deletion mutant lacking the *STE20* or *STE11* gene involved in the activation of the MAPK cascade by using NMY51 as the parental strain with an aim to enable screening of GPCR dimers with and without ligand (**Fig. 1** **and** **Table 1**). Halo bioassays responding to α-factor pheromone showed the formations of a thin halo and no halos with 100 µg of α-factor in NMY61 (*ste20*Δ) and NMY62 (*ste11*Δ) yeast strains, respectively, revealing that *ste11*Δ allele provides more strict avoidance of signal-promoted growth arrest in the presence of ligand (**Fig. 2A**). For checking the expressions of reporter genes, the growth assays in the presence of ligand were carried out (**Fig. 2B**). While the strains harboring mock vectors pBT3-C and pPR3-C were used as negative controls, those harboring pCCW-Alg5 and pAI-Alg5 to express Alg5-Nub*I* and Alg5-Cub-LexA-VP16 were used as positive controls. Alg5-Nub*I* is a yeast membrane protein fused with a WT Nub tag. The Nub*I* tag interacts spontaneously with any Cub tag-containing constructs [11], [12]. The deletion mutants (*ste20*Δ and *ste11*Δ) avoided the robust growth inhibition and therefore could allow the growth assays with *ADE2* and *HIS3* reporter genes even in the presence of ligand (**Fig. 2B**). We used the MAPK-defective NMY62 yeast strain for the following experiments.

**Table 1:** Yeast strains used in this study.

Strain	Genotype	Source
NMY51	*MAT* ***a*** * his3*Δ*200 trp1-901 leu2-3, 112 ade2 LYS2*::*(lexAop)_4_-HIS3 ura3*::*(lexAop)_8_-lacZ ade2*::*(lexAop)_8_-ADE2 GAL4*	Dualsystems Biotech AG
NMY61	NMY51 *ste20*Δ	This study
NMY62	NMY51 *ste11*Δ	This study
NMY63	NMY51 *ste11*Δ *ste2*Δ	This study

To test the viability of split-ubiquitin–based reporter gene assays for detecting GPCR dimers, we first analyzed the homodimerization of endogenous yeast pheromone receptor (Ste2p) in the NMY62 yeast strain. The N-terminal moiety of split-ubiquitin with an I13G mutation (Nub*G*) and the C-terminal ubiquitin moiety linked to an artificial transcription factor (Cub-LexA-VP16) [7] were respectively designed to genetically fuse to the C-termini of Ste2p receptors by using original pPR3-C (prey) and pBT3-C (bait) split-ubiquitin vectors (**Table S2**). Upon *in vivo* protein-protein interaction, the reconstituted ubiquitin leads to cleavage and release of LexA-VP16 by ubiquitin-specific proteases (UBPs) [7]; therefore, the dimerization of Ste2p should be detected via the transcription activation of the reporter genes (*ADE2*, *HIS3*, and *lacZ*) (**Fig. 1** **and** **Table 1**). However, the cells coexpressing Ste2p-Nub*G* and Ste2p-Cub-LexA-VP16 never grew on the adenine/histidine-deficient selectable media (**Fig. S1A**). Therefore, we replaced the weak *CYC1* promoter of the original pBT3-C bait vector by comparatively strong *PHO5*, *TPI1* and *TDH3* promoters (*P_CYC1_*<*P_PHO5_*<*P_TPI1_*<*P_TDH3_*). As a result, the expression of Ste2p-Cub-LexA-VP16 by the *TPI1* and *TDH3* promoters prompted cell growth on the selection media when combined with the expression of Ste2p-Nub*G* (**Fig. S1B and C**). Even though previous report expressed the Ste2p in relatively low expression manner [13], our result did not show evidence of dimerization for the Ste2p expressed under the control of the *CYC1* promoter in the split-ubiquitin system. This is assumed that attached transcription factor might be not present at levels adequate to activate the response.

**Figure 3:**
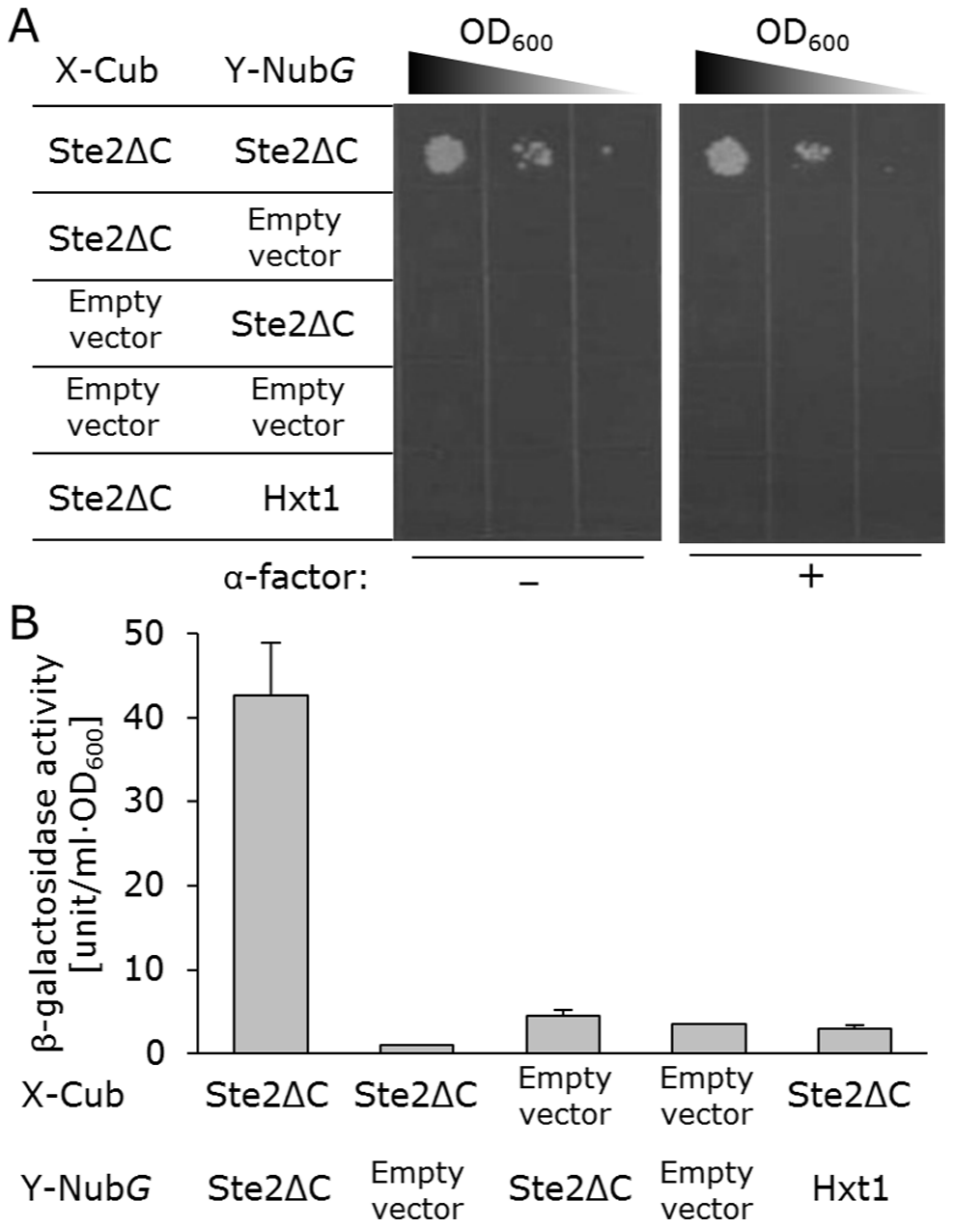
Detection for dimerization of yeast truncated Ste2p lacking the carboxy-terminal tail (Ste2ΔC) receptor in NMY62 strain. Growth and quantitative β-galactosidase activity of yeast cells expressing various combinations of Cub and Nub*G* fusions. The control bait plasmid was pBT3-C mock vector (empty vector). The control prey plasmids were pPR3-C mock vector (empty vector) and pPR3-HXT1. (A) Growth assay without α-factor (*left panels*) and with 5 µM α-factor (*right panels*). Each cell was spotted in serial 10-fold dilutions on SD –Leu, Trp, Ade and His dropout plate. (B) Quantitative β-galactosidase assay. Error bars represent the standard deviations (*n = *3).

Then, we replaced the full-length Ste2p receptors with a truncated version that lacks the C-terminal tails (Ste2ΔC; amino acids 1–304) to adjust the distance between the C-termini of the receptors [13], providing an increased signal-to-noise (S/N) ratio (under the control of the *TPI1* promoter; **Fig. S2A and B**). Furthermore, we replaced the *TPI1* promoter with the *CYC1* promoter of the bait vector again, which resulted in much lower background cell growth and a drastically improved S/N ratio (**Fig. 3A and B**). Since the truncation of the C-termini of Ste2p receptors had been reported to increase in the number of receptor sites [14], the greatly enriched receptors at the plasma membrane might have provided the drastic improvement of S/N ratio. These results indicate that the bait receptor is predominant for successful detection of the dimerized receptors. Thus, the consideration of the receptor’s expression manner is important to screen the GPCR dimer partners, because the leaky background cell growth often brings unanticipated candidates.

**Figure 4:**
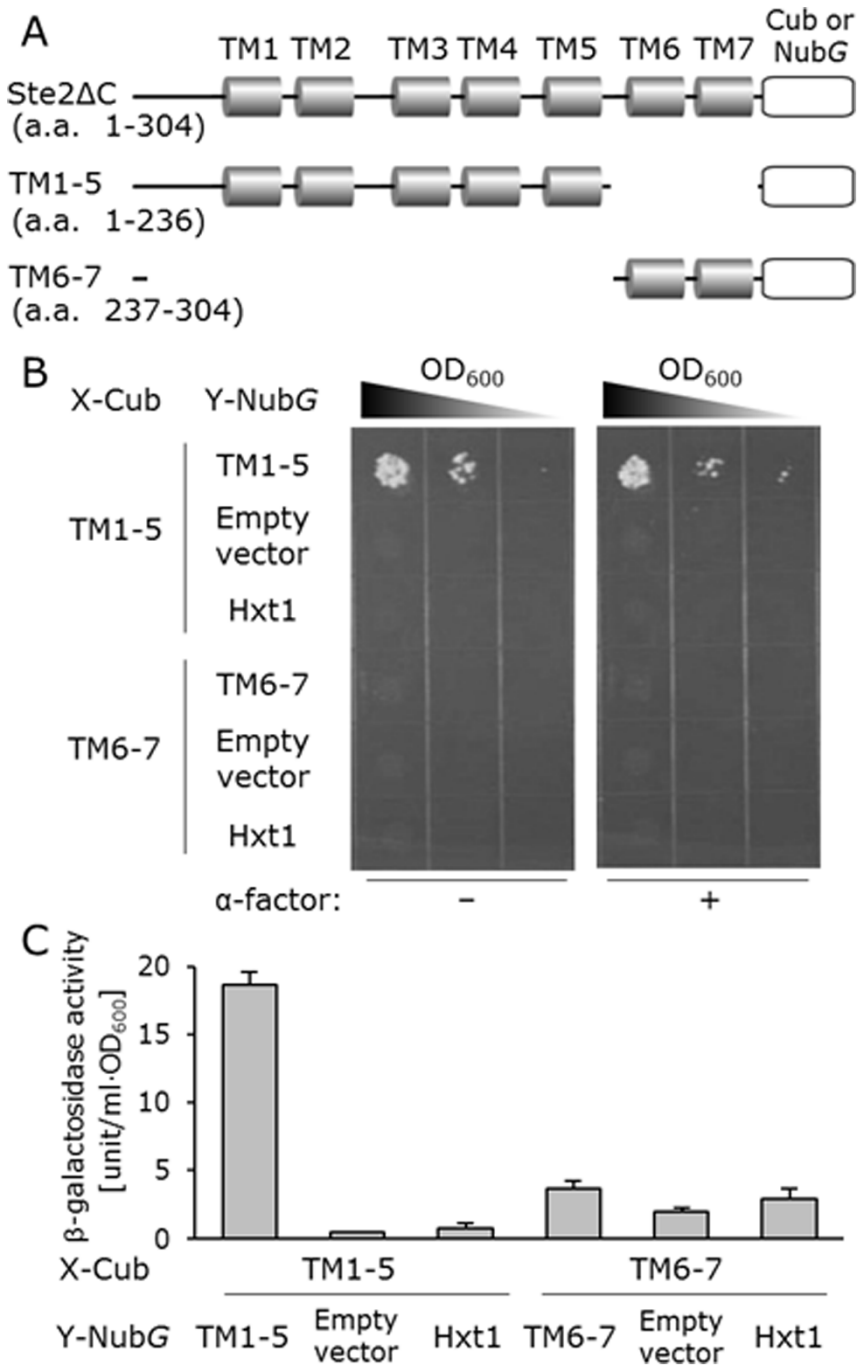
Detection for dimerization of yeast Ste2p deletion mutants (TM1–5 and TM6–7) in NMY62 strain. (A) Schematic of Ste2ΔC and the deletion mutants. Transmembrane (TM) domains are indicated with pillar-type boxes, and the Cub (Cub-LexA-VP16) or Nub*G* (Nub with I13G mutation) is depicted as a rounded rectangle. (B) Growth assay without α-factor (*left panels*) and with 5 µM α-factor (*right panels*). Each cell was spotted in serial 10-fold dilutions on SD –Leu, Trp, Ade and His dropout plate. (C) Quantitative β-galactosidase activity in yeast cells containing various combinations of plasmids. Error bars represent the standard deviations (*n = *3). The control prey plasmids were pPR3-C mock vector (empty vector) and pPR3-HXT1.

In the optimized system, Hxt1p never presented background cell growth in the dimerization assay with the Ste2ΔC receptor (**Fig. 3A**). Previously, Overton et al. had used the Hxt1p as the negative control for FRET analysis of Ste2p dimerization and confirmed the subcellular localization of it at the plasma membrane [15]. The β-galactosidase assay that reflects *lacZ* reporter enzyme activity also displayed similar trends (**Fig. 3B**). The addition of ligand had no effect on the dimerization events of Ste2ΔC, since the MAPK-defective NMY62 yeast strain displayed unchanged growth in the presence of α-factor (**Fig. 3A**). We additionally constructed two types of deletion mutants in which the TM6–7 domains and the TM1–5 domains were removed, respectively (TM1–5 (amino acids 1–236) and TM6–7 (amino acids 237–304)) (**Fig. 4A**). Overton et al. also indicated that self-association of TM6–7 had not been detected, and plasma membrane localization of the YFP-tagged TM1–5 and TM6–7 was observed [16]. As previously indicated [16], only TM1–5 formed the homodimer (**Fig. 4B and C**), showing that our system is applicable to examine critical domains involved in the dimerization of 7TM receptors.

**Figure 5:**
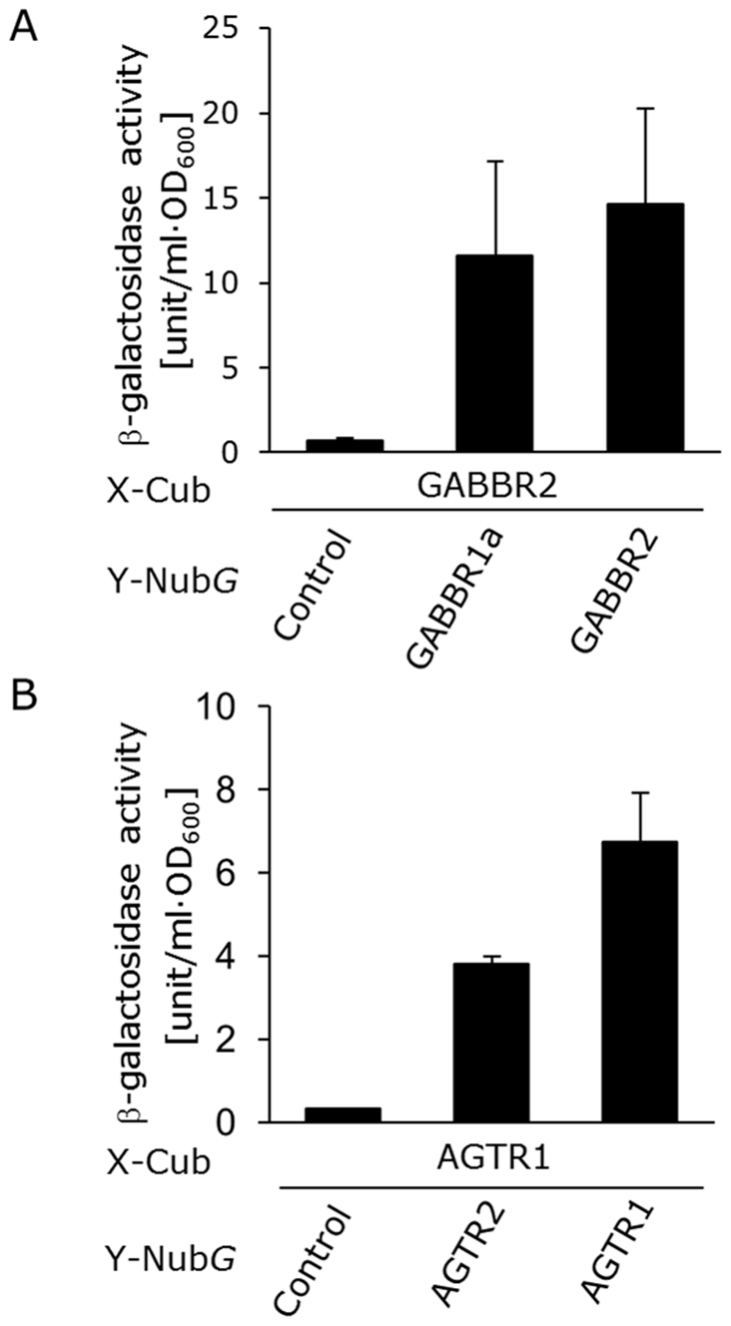
Dimerization assays of human GPCRs. Quantitative β-galactosidase assay. (A) Detection of GABA_B1a_/GABA_B2_ (GABBR1a/GABBR2) heterodimers. (B) Detection of AT_1_/AT_2_ (AGTR1/AGTR2) heterodimers. Error bars represent the standard deviations (*n = *3). The control prey plasmid was pPR3-C mock vector.

Subsequently, we validated the capability of our system to detect human GPCR heterodimer pairs. To avoid competitive dimerization with the endogenous yeast Ste2p receptor, we constructed an NMY63 mutant strain in which the *STE2* gene was additionally deleted (**Table 1** **and Fig. S3**). Since clear evidence for the functional role of GPCR homodimer and heterodimer pairs was first obtained for class C receptors, such as GABA_B_ receptors [17], we tested the dimerization of GABA_B2_ receptor (GABBR2) with GABA_B1a_ receptor (GABBR1a). The β-galactosidase assay clearly showed the specific activities both for GABA_B2_/GABA_B2_ and GABA_B2_/GABA_B1a_ couples (**Fig. 5A** **and Fig. S4A**). This result was coincident with the fact that GABA_B2_ receptor could form not only heterodimer with GABA_B1a_ receptor but also homodimer [18], [19], indicating that the split-ubiquitin-based approach could detect the homodimerization and heterodimerization of GABA_B2_ receptors. Additionally, the β-galactosidase assay for dimerization of GABA_B1a_ receptor also showed similar results (**Fig. S5**).

In contrast to the widely accepted concept of class C GPCR dimerization, the significance of *in vivo* dimerization of class A GPCRs remains controversial [20]. Since a growing amount of evidence indicates that class A GPCRs are able to form dimers or higher-ordered oligomers *in vivo* [21], [22], we next evaluated class A GPCR heterodimer pairs. As class A GPCRs, AT_1_ and AT_2_ angiotensin receptors (AGTR1 and AGTR2) were selected. Consistent with previous reports [23], [24], the β-galactosidase assay also illustrated the formation of heterodimers between AGTR1 and AGTR2 (**Fig. 5B** **and Fig. S4B**).

**Figure 6:**
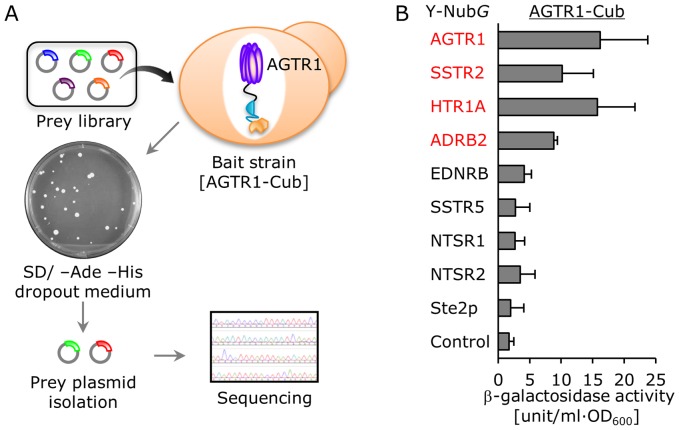
Screening of candidate heterodimer partners of AT_1_ angiotensin receptor (AGTR1). (A) Workflow of a yeast two-hybrid screen. Prey library was transformed into the NMY63 yeast strains harboring AGTR1 bait vector, and the selection with growth reporter genes was performed. Following isolation of prey plasmids from each colony, the obtained GPCR clones were determined by sequencing analysis. (B) Quantitative β-galactosidase assays for homo- and hetero-dimerization of AGTR1 in NMY63 strain. NMY63 yeast strain was transformed with GPCR-Nub*G* indicated at the left and AGTR1-Cub-LexA-VP16. The control prey plasmid was pPR3-C mock vector. Error bars represent the standard deviations (*n = *3).

Next, we aimed to apply our system to screen new candidate heterodimer partners of AGTR1 receptor within the class A GPCRs, except for AGTR2. The *CYC1* promoter was selected for expressing AGTR1 as a bait protein (**Fig. S4B**). As a model candidate library, we constructed the prey vectors to express AGTR1 (as a positive control), β_2_-adrenergic receptor (ADRB2), 5-hydroxytryptamine (serotonin) receptor 1A (HTR1A), somatostatin receptor 2 (SSTR2), somatostatin receptor 5 (SSTR5), endothelin receptor type B (EDNRB), neurotensin receptor 1 (NTSR1) and neurotensin receptor 2 (NTSR2) (**Table S2 and S3**) and then mixed equal amounts of these 9 prey vectors (containing pPR3-C mock vector). After introduction of the constructed library into the NMY63 yeast strains harboring AGTR1 bait vector, the selection with *ADE2*/*HIS3* growth reporter genes was performed (**Fig. 6A**). A total of 30 colonies was generated on the adenine/histidine-deficient selection media (**Fig. 6A**). Following isolation of prey plasmids from each colony, the obtained GPCR clones were determined by sequencing analysis. Ten clones of AGTR1 were dominantly identified as the homodimer (33.3%), whereas 5 clones of SSTR2 (16.7%), 3 clones of ADRB2 (10.0%) and 3 clones of HTR1A (10.0%) were successfully screened as the candidate heterodimer partners for AGTR1.

**Figure 7:**
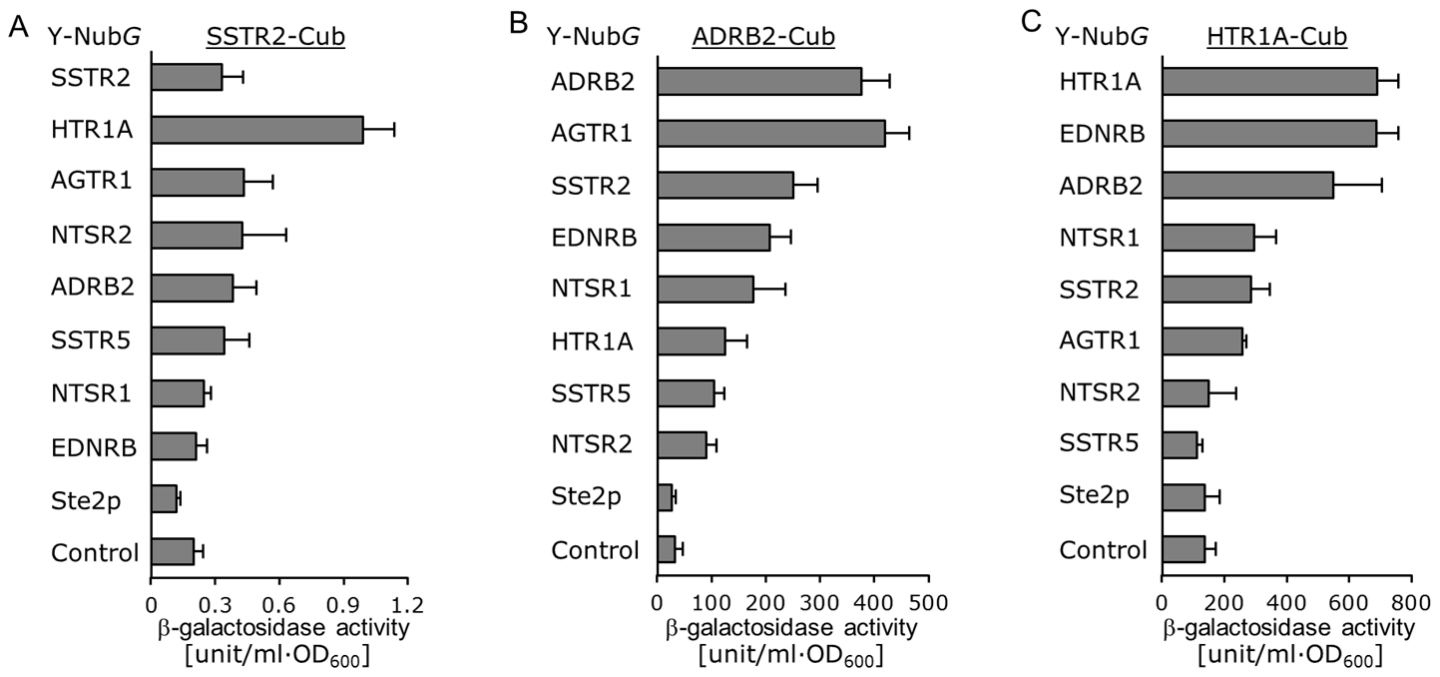
Quantitative β-galactosidase assays for homo- and hetero-dimerization between human-GPCRs in NMY63 strain. NMY63 yeast strain was transformed with GPCR-Nub*G* indicated at the left and SSTR2-Cub-LexA-VP16 (A), ADRB2-Cub-LexA-VP16 (B), or HTR1A-Cub-LexA-VP16 (C). The control prey plasmid was pPR3-C mock vector. Error bars represent the standard deviations (*n = *3).

To validate the success or failure of the screening, we measured the β-galactosidase activities of the yeast cells separately co-transformed with the AGTR1 bait vector and 9 other prey vectors including the previously reported AGTR1/ADRB2 heterodimer pairs [25], yeast Ste2p control receptor and mock control. The results likely reflected the occupancies of identified clones, indicating that our system succeeded in screening heterodimer candidates (**Fig. 6B**). Additionally, β-galactosidase activities measured with other GPCRs as bait proteins were fairly consistent with the results of the screening and also revealed new candidates for heterodimer pairs including SSTR2/HTR1A, SSTR2/ADRB2, and HTR1A/EDNRB (**Fig. 7A–C** **and Fig. S4C–E**). Our experiments indicated that Ste2p could not co-oligomerize with the human GPCRs (**Fig. 6B** **and** **Fig. 7A–C**).

**Figure 8:**
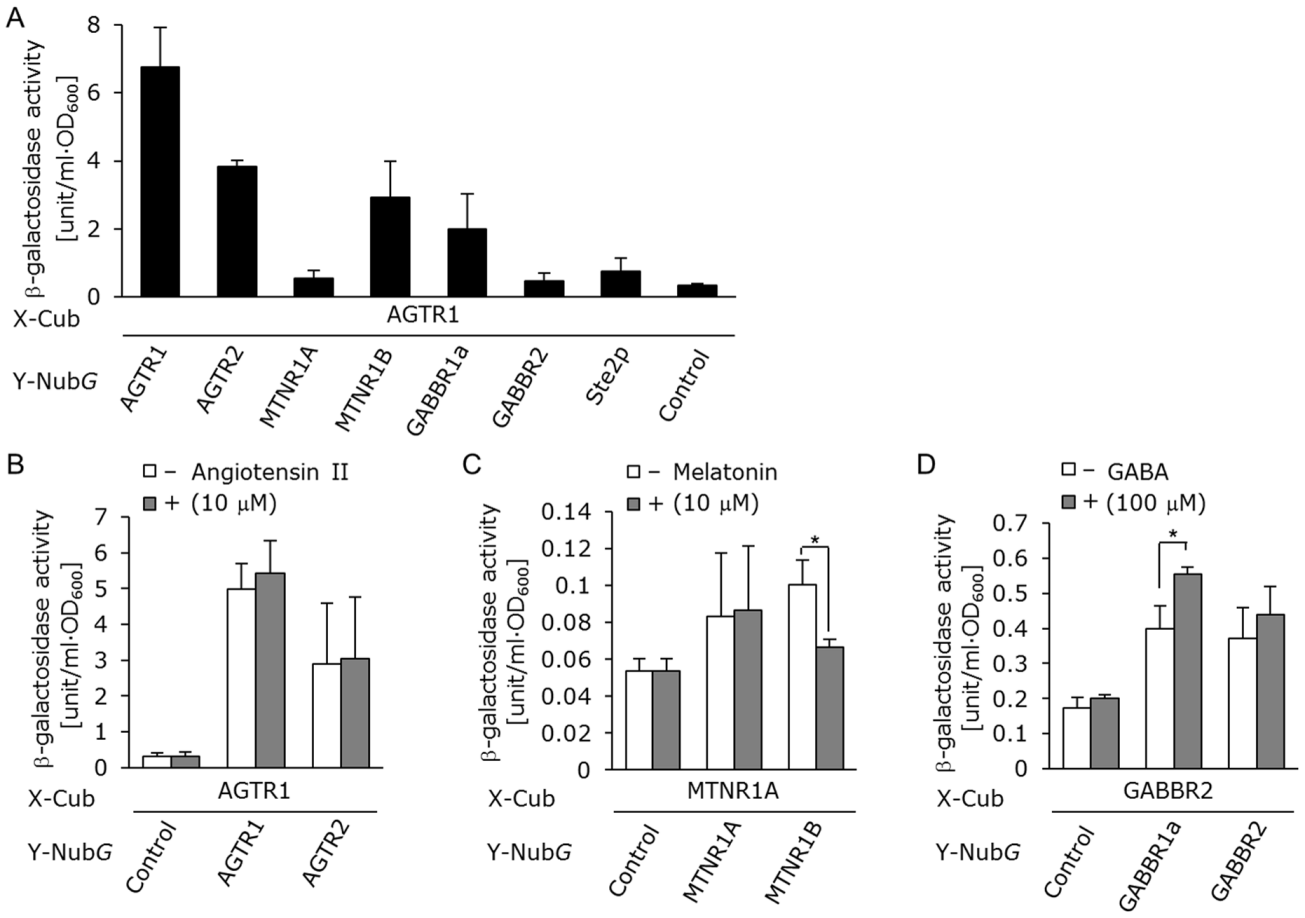
Detection for dimerization and ligand-induced conformational changes of human GPCRs. (A) Quantitative β-galactosidase assays for heterodimerization of AGTR1 in NMY63 strain. NMY63 yeast strain was transformed with GPCR-Nub*G* indicated at the bottom and AGTR1-Cub-LexA-VP16. (B–D) Ligand assays for detection of conformational changes in GPCR dimerizations. (B) AT_1_/AT_2_ (AGTR1/AGTR2) heterodimers. Incubation time, 18 h. Angiotensin II conc., 0 or 10 µM. (C) MT_1_/MT_2_ (MTNR1A/MTNR1B) heterodimers. Incubation time, 18 h. Melatonin conc., 0 or 10 µM. (D) GABA_B1a_/GABA_B2_ (GABBR1a/GABBR2) heterodimers. Incubation time, 18 h. GABA conc., 0 or 100 µM. The control prey plasmid was pPR3-C mock vector. Error bars represent the standard deviations (*n = *3). (*P<0.05).

Additionally, we measured the β-galactosidase activities of the yeast cells separately co-transformed with the AGTR1 bait vector and GABBR1a, GABBR2, MT_1_ and MT_2_ melatonin receptor (MTNR1A and MTNR1B) prey vectors. The results indicated new candidates for heterodimer pairs including AGTR1/GABBR1a and AGTR1/MTNR1B (**Fig. 8A**). Thus, the obtained results from all heterodimerization assays with the split-ubiquitin system might have implicated a general statement about the ability of various human GPCRs to heterooligomerize with each other.

Finally, we performed detection of not only the dimer formation of target human GPCRs but also the ligand-mediated conformational changes in living yeast cells. In the case of AGTR1 the addition of 10 µM of native ligand, angiotensin II, did not affect the states of the homodimerized and heterodimerized receptors with AGTR2 (**Fig. 8B**). MT_1_ and MT_2_ melatonin receptors (MTNR1A and MTNR1B) not only form heterodimers, but also induce a conformational change within the heterodimers [4]. In addition, it has been reported that expressions of MTNR1A and MTNR1B in yeast activated the pheromone signaling pathway via the endogenous yeast G-proteins in response to the native ligand melatonin [26], [27]. β-galactosidase assays based on the split-ubiquitin technique in the MAPK-defective NMY63 yeast strain allowed successful detection of the conformational change of MTNR1A/MTNR1B heterodimers in the presence of melatonin (**Fig. 8C** **and Fig. S4F**), suggesting that our system can detect ligand-mediated conformational changes as well as the heterodimer formations. Moreover, we also tested the detection of the conformational change of GABA_B2_/GABA_B1a_ heterodimers in the presence of GABA (**Fig. 8D**). While the addition of 100 µM of GABA did not affect the states of the GABA_B2_ homodimers, the β-galactosidase assay exposed the conformational change of GABA_B2_/GABA_B1a_ heterodimers in consistency with previous reports [28] (**Fig. 8D**).

Because a positive result in the assay potentially could come about through indirect association of receptors via a third protein or close co-localization, it is important to re-evaluate the irrefragability with other methods or in human cells. Additionally, there is no guarantee that association is actually physiologically relevant. However, it is also true that this system could narrow down the new candidates of GPCR heterodimers with a bit of effort. If one accepts these uncertainties, the assay provides a nice way of monitoring changes that does not depend on effective downstream signaling through the GPCR pathway.

In summary, we have developed a specialized method to screen candidate heterodimer partners for target GPCRs based on the split-ubiquitin membrane yeast two-hybrid method. This modified system permitted the rapid and facile detection of not only the heterodimer formation of target human GPCRs, but also the ligand-mediated conformational changes in living yeast cells. Since budding yeast *Saccharomyces cerevisiae* can functionally express human GPCRs [29], [30], construction of a large prey library would be beneficial for the identification of heterodimer candidates as the partners of target human GPCRs. Our system will be a useful tool to assist in the intermolecular mapping of interactions among GPCRs and uncover potential targets for the development of new therapeutic agents.

## Materials and Methods

### Media

Synthetic dextrose (SD) media contained 6.7 g/l yeast nitrogen base without amino acids (YNB) (BD Diagnostic Systems, Sparks, MD, USA) and 20 g/l glucose. YPDA media contained 10 g/l yeast extract, 20 g/l peptone, 20 g/l glucose and 55 mg/l adenine. Amino acids and nucleotides (60 mg/l leucine, 40 mg/l tryptophan, 40 mg/l adenine, 20 mg/l histidine or 20 mg/l uracil) were supplemented into SD media to provide the relevant auxotrophic components. For solid plates, 2% agar was added to the media.

### Yeast Strains

All yeast strains were generated from NMY51 (Dualsystems Biotech AG, Schlieren, Switzerland) as a parental backbone strain and are listed in **Table 1**. Transformation with linear DNA fragments was performed by using the lithium acetate method [31]. To eliminate the *URA3* selectable marker in each transformation step, we basically followed previous procedures [32], [33] with the marker recycling method [34]. All oligonucleotides used for the strain constructions are listed in **Table S1**. To disrupt the target genes (*STE20*, *STE11* and *STE2*), the first half of DNA fragments containing upstream regions of target genes and *URA3* selectable marker were PCR-amplified from pGK406 [35] by using gene-specific oligonucleotides. The last half of DNA fragments containing downstream regions of target genes and homologous sequences to eliminate *URA3* marker were PCR-amplified from NMY51 genomic DNA by using gene-specific oligonucleotides. These amplified fragments were then used as the templates for overlap PCR. The combined linear fragments were introduced into appropriate parental yeast strains, and the transformants were selected on SD solid media lacking uracil. After confirming integration of the fragments at the correct positions, the cells were maintained on SC media containing 1 mg/ml 5-fluoroorotic acid (5-FOA, Fluorochem, Derbyshire, UK) to eliminate *URA3* marker.

### Plasmids

Plasmid construction is described in **Document S1**. All plasmids used for the assays are listed in **Table S2**. The transformation procedure followed the lithium acetate method [31].

### Agar Diffusion Bioassay

An agar diffusion bioassay (halo assay) was performed to measure growth inhibition in response to signal-induced cell-cycle arrest [36]. Cells were grown in YPDA media overnight at 30°C. Sterilized paper filter disks (6 mm in diameter) were placed on a square Petri dish, and various amounts of α-factor pheromone (Zymo Research, Orange, CA, USA) were spotted onto the disks. YPDA medium containing 20 g/l agar (maintained at 50°C) was inoculated with the grown cells to give an initial optical density of 5×10^−4^ at 600 nm (OD_600_ = 5×10^−4^), and the suspension was immediately poured into the dish. The plates were incubated at 30°C for 1 to 2 days.

### Evaluation of Receptor Dimerization

Cub and Nub*G* fusion constructs (**Table S2**) were co-transformed into yeast strains. Cells were grown in SD media lacking leucine and tryptophan overnight at 30°C on a rotatory shaker set at 150 rpm and then harvested to evaluate receptor dimerization by growth assay and β-galactosidase assay.

### Growth Assay

Harvested cells were washed with distilled water, and cell suspensions were prepared to give an OD_600_ of 10. Seven microliters of serial dilutions of cell suspensions (1∶10) were spotted on SD agar plates lacking leucine, tryptophan, adenine and histidine. The plates were incubated at 30°C.

### β-D-galactosidase Activity Assay

β-D-galactosidase activity was determined by using chlorophenol red β-D-galactopyranoside (CPRG) (Roche Applied Science, Indianapolis, IN, USA) as the chromogenic substrate. The procedure basically followed the method described in the Yeast Protocols Handbook (Clontech Laboratories, Inc./Takara Bio Company). Harvested cells were washed once with buffer 1 [100 mM 4-(2-hydroxyethyl)-1-piperazineethanesulfonic acid (HEPES), 150 mM NaCl, 4 mM L-aspartic acid hemimagnesium salt hydrate, 10 g/l bovine serum albumin (BSA) and 0.05% polyoxyethylene sorbitan monolaurate (Tween 20); pH 7.25–7.30] and resuspended in buffer 1 to give an OD_600_ of 10. Four microliters of chloroform and 7 µl of 0.1% SDS were added to 100 µl of cell suspension, the mixtures were agitated with a vortex, and then buffer 1 (700 µl) containing 2.23 mM CPRG was added to the mixtures. After incubation for 10 min at room temperature, 500 µl of 3 mM ZnCl_2_ was added to stop the enzyme reaction. After centrifugation, the OD_578_ of supernatants were measured with a spectrophotometer. β-Gal units were calculated as 1,000×OD_578_/(10 min×0.1 ml×OD_600_).

### Ligand Assay

Harvested cells were inoculated into 5 mL of fresh SD media containing ligand to give an initial OD_600_ of 0.03. They were incubated at 30°C with shaking at 150 rpm for up to 18 h. Afterwards, the β-D-galactosidase activity was performed.

### Model Screening

A small-sized prey GPCR library (**Table S3**) was transformed into yeast strain NMY63 harboring pBT3-AGTR1 by using the lithium acetate method [31]. Transformants were selected on SD medium lacking leucine, tryptophan, adenine and histidine for bait-prey interaction. Prey plasmids were isolated from 30 positive clones, amplified in *Escherichia coli*, and analyzed by sequencing analysis.

## Supporting Information

Figure S1 **Effect of promoter on detection of dimerization of yeast Ste2p receptor in NMY62 strain.** NMY62 yeast strain was transformed with the plasmids expressing indicated protein pairs and grown on SD –Leu, Trp, Ade and His dropout plate at 30°C. (A) *CYC1* promoter. (B) *PHO5* promoter and *TPI1* promoter. (C) *TDH3* promoter.
(TIF)

Figure S2 **Effect of removal of C-terminal tail on detection of dimerization of yeast Ste2p receptor in NMY62 strain.** NMY62 yeast strain was transformed with plasmids expressing indicated protein pairs. Quantitative β-galactosidase assays for full-length Ste2p receptor under the control of *TPI1* promoter (A) and Ste2p receptor that lacks the C-terminal tail (Ste2ΔC) under the control of *TPI1* promoter (B).
(TIF)

Figure S3 **Detection of homodimerization of human SSTR5 receptor in NMY62 or NMY63 strains.** NMY63 yeast strain was transformed with plasmids expressing indicated protein pairs. Quantitative β-galactosidase assays for the NMY62 strain (A) and NMY63 strain (B).
(TIF)

Figure S4 **Optimization of promoter to detect homodimerization of human GPCRs in NMY63 strain.** NMY63 yeast strain was transformed with plasmids expressing the indicated protein pairs. (A) Quantitative β-galactosidase assays. (B–F) Growth assays on SD –Leu, Trp, Ade and His dropout plate at 30°C. (A) GABA_B2_ receptor (GABBR2) (B) AT_1_ angiotensin receptor (AGTR1) (C) MT_1_ melatonin receptor (MTNR1A) (D) somatostatin receptor 2 (SSTR2) (E) β_2_-adrenergic receptor (ADRB2) (F) 5-hydroxytryptamine (serotonin) receptor 1A (HTR1A). The control prey plasmid was pPR3-C mock vector.
(TIF)

Figure S5 **Detection of homo- and hetero-dimerization of human GABA_B1a_ receptor with GABA_B2_ receptor in NMY63 strain.** Quantitative β-galactosidase assay. NMY63 yeast strain was transformed with plasmids expressing indicated protein pairs. Error bars represent the standard deviations (*n* = 3). The control prey plasmid was pPR3-C mock vector.
(TIF)

Table S1 List of oligonucleotides.
(PDF)

Table S2 List of plasmids.
(PDF)

Table S3 List of prey GPCR library.
(PDF)

Document S1 **Supplementary Materials and Methods (Plasmid constructions for supporting information)**.
(PDF)

## References

[1] Panetta R, Greenwood MT (2008) Physiological relevance of GPCR oligomerization and its impact on drug discovery. Drug Discov Today 13: 1059–1066. 18824244 10.1016/j.drudis.2008.09.002

[2] George SR, O’Dowd BF, Lee SP (2002) G-protein-coupled receptor oligomerization and its potential for drug discovery. Nat Rev Drug Discov 1: 808–820. 12360258 10.1038/nrd913

[3] Percherancier Y, Berchiche YA, Slight I, Volkmer-Engert R, Tamamura H, et al (2005) Bioluminescence resonance energy transfer reveals ligand-induced conformational changes in CXCR4 homo- and heterodimers. J Biol Chem 280: 9895–9903. 15632118 10.1074/jbc.M411151200

[4] Ayoub MA, Levoye A, Delagrange P, Jockers R (2004) Preferential formation of MT1/MT2 melatonin receptor heterodimers with distinct ligand interaction properties compared with MT2 homodimers. Mol Pharmacol 66: 312–321. 15266022 10.1124/mol.104.000398

[5] Fredriksson R, Lagerström MC, Lundin LG, Schiöth HB (2003) The G-protein-coupled receptors in the human genome form five main families. Phylogenetic analysis, paralogon groups, and fingerprints. Mol Pharmacol 63: 1256–1272. 12761335 10.1124/mol.63.6.1256

[6] Pfleger KD, Eidne KA (2005) Monitoring the formation of dynamic G-protein-coupled receptor-protein complexes in living cells. Biochem J 385: 625–637. 15504107 10.1042/BJ20041361 PMC1134737

[7] Stagljar I, Korostensky C, Johnsson N, te Heesen S (1998) A genetic system based on split-ubiquitin for the analysis of interactions between membrane proteins in vivo. Proc Natl Acad Sci USA 95: 5187–5192. 9560251 10.1073/pnas.95.9.5187 PMC20236

[8] Ishii J, Fukuda N, Tanaka T, Ogino C, Kondo A (2010) Protein–protein interactions and selection: yeast-based approaches that exploit guanine nucleotide-binding protein signaling. FEBS J 277: 1982–1995. 22428173 10.1111/j.1742-4658.2010.07625.x

[9] Elion EA, Satterberg B, Kranz JE (1993) FUS3 phosphorylates multiple components of the mating signal transduction cascade: evidence for STE12 and FAR1. Mol Biol Cell 4: 495–510. 8334305 10.1091/mbc.4.5.495 PMC300953

[10] Ishii J, Tanaka T, Matsumura S, Tatematsu K, Kuroda S, et al (2008) Yeast-based fluorescence reporter assay of G protein-coupled receptor signalling for flow cytometric screening: FAR1-disruption recovers loss of episomal plasmid caused by signalling in yeast. J Biochem 143: 667–674. 18281298 10.1093/jb/mvn018

[11] Iyer K, Bürkle L, Auerbach D, Thaminy S, Dinkel M, et al (2005) Utilizing the split-ubiquitin membrane yeast two-hybrid system to identify protein-protein interactions of integral membrane proteins. Sci STKE 2005: pl3. 15770033 10.1126/stke.2752005pl3

[12] Kittanakom S, Chuk M, Wong V, Snyder J, Edmonds D, et al (2009) Analysis of membrane protein complexes using the split-ubiquitin membrane yeast two-hybrid (MYTH) system. Methods Mol Biol 548: 247–271. 19521829 10.1007/978-1-59745-540-4_14

[13] Gehret AU, Bajaj A, Naider F, Dumont ME (2006) Oligomerization of the yeast alpha-factor receptor: implications for dominant negative effects of mutant receptors. J Biol Chem 281: 20698–20714. 16709573 10.1074/jbc.M513642200

[14] Konopka JB, Jenness DD, Hartwell LH (1988) The C-terminus of the S. cerevisiae alpha-pheromone receptor mediates an adaptive response to pheromone. Cell 54: 609–620. 2842059 10.1016/s0092-8674(88)80005-9

[15] Overton MC, Blumer KJ (2000) G-protein-coupled receptors function as oligomers in vivo. Curr Biol 10: 341–344. 10744981 10.1016/s0960-9822(00)00386-9

[16] Overton MC, Blumer KJ (2002) The extracellular N-terminal domain and transmembrane domains 1 and 2 mediate oligomerization of a yeast G protein–coupled receptor. J Biol Chem 277: 41463–41472. 12194975 10.1074/jbc.M205368200

[17] Kaupmann K, Malitschek B, Schuler V, Heid J, Froestl W, et al (1998) GABA(B)–receptor subtypes assemble into functional heteromeric complexes. Nature 396: 683–687. 9872317 10.1038/25360

[18] Maurel D, Kniazeff J, Mathis G, Trinquet E, Pin JP, et al (2004) Cell surface detection of membrane protein interaction with homogeneous time-resolved fluorescence resonance energy transfer technology. Anal Biochem 329: 253–262. 15158484 10.1016/j.ab.2004.02.013

[19] Maurel D, Comps-Agrar L, Brock C, Rives ML, Bourrier E, et al (2008) Cell-surface protein-protein interaction analysis with time-resolved FRET and snap-tag technologies: application to GPCR oligomerization. Nat Methods 5: 561–567. 18488035 10.1038/nmeth.1213 PMC2642604

[20] Prezeau L, Rives ML, Comps–Agrar L, Maurel D, Kniazeff J, et al (2010) Functional crosstalk between GPCRs: with or without oligomerization. Curr Opin Pharmacol 10: 6–13. 19962942 10.1016/j.coph.2009.10.009

[21] Rivero-Müller A, Chou YY, Ji I, Lajic S, Hanyaloglu AC, et al (2010) Rescue of defective G protein-coupled receptor function in vivo by intermolecular cooperation. Proc Natl Acad Sci USA 107: 2319–2324. 20080658 10.1073/pnas.0906695106 PMC2836644

[22] Albizu L, Cottet M, Kralikova M, Stoev S, Seyer R, et al (2010) Time-resolved FRET between GPCR ligands reveals oligomers in native tissues. Nat Chem Biol 6: 587–594. 20622858 10.1038/nchembio.396 PMC3506176

[23] Lyngsø C, Erikstrup N, Hansen JN (2009) Functional interactions between 7TM receptors in the renin-angiotensin system–dimerization or crosstalk? Mol Cell Endocrinol 302: 203–212. 18930783 10.1016/j.mce.2008.09.018

[24] Porrello ER, Pfleger KD, Seeber RM, Qian H, Oro C, et al (2011) Heteromerization of angiotensin receptors changes trafficking and arrestin recruitment profiles. Cell Signal 23: 1767–1776. 21740964 10.1016/j.cellsig.2011.06.011

[25] Barki-Harrington L, Luttrell LM, Rockman HA (2003) Dual inhibition of beta-adrenergic and angiotensin II receptors by a single antagonist: a functional role for receptor-receptor interaction in vivo. Circulation 108: 1611–1608. 12963634 10.1161/01.CIR.0000092166.30360.78

[26] Kokkola T, Watson MA, White J, Dowell S, Foord SM, et al (1998) Mutagenesis of human Mel1a melatonin receptor expressed in yeast reveals domains important for receptor function. Biochem Biophys Res Commun 249: 531–536. 9712731 10.1006/bbrc.1998.9182

[27] Brown AJ, Dyos SL, Whiteway MS, White JH, Watson MA, et al (2000) Functional coupling of mammalian receptors to the yeast mating pathway using novel yeast/mammalian G protein α-subunit chimeras. Yeast 16: 11–22. 10620771 10.1002/(SICI)1097-0061(20000115)16:1<11::AID-YEA502>3.0.CO;2-K

[28] Matsushita S, Nakata H, Kubo Y, Tateyama M (2010) Ligand-induced rearrangements of the GABA(B) receptor revealed by fluorescence resonance energy transfer. J Biol Chem 285: 10291–10299. 20129919 10.1074/jbc.M109.077990 PMC2856234

[29] Li B, Scarselli M, Knudsen CD, Kim SK, Jacobson KA, et al (2007) Rapid identification of functionally critical amino acids in a G protein-coupled receptor. Nat Methods 4: 169–174. 17206152 10.1038/nmeth990

[30] Ishii J, Yoshimoto N, Tatematsu K, Kuroda S, Ogino C, et al (2012) Cell wall trapping of autocrine peptides for human G-protein-coupled receptors on the yeast cell surface. PLoS One 7: e37136. 22623985 10.1371/journal.pone.0037136 PMC3356411

[31] Gietz D, St Jean A, Woods RA, Schiestl RH (1992) Improved method for high efficiency transformation of intact yeast cells. Nucleic Acids Res 20: 1425. 1561104 10.1093/nar/20.6.1425 PMC312198

[32] Togawa S, Ishii J, Ishikura A, Tanaka T, Ogino C, et al (2010) Importance of asparagine residues at positions 13 and 26 on the amino-terminal domain of human somatostatin receptor subtype-5 in signalling. J Biochem 147: 867–873. 20207824 10.1093/jb/mvq022

[33] Iguchi Y, Ishii J, Nakayama H, Ishikura A, Izawa K, et al (2010) Control of signalling properties of human somatostatin receptor subtype-5 by additional signal sequences on its amino-terminus in yeast. J Biochem 147: 875–884. 20207822 10.1093/jb/mvq023

[34] Akada R, Kitagawa T, Kaneko S, Toyonaga D, Ito S, et al (2006) PCR-mediated seamless gene deletion and marker recycling in Saccharomyces cerevisiae. Yeast 23: 399–405. 16598691 10.1002/yea.1365

[35] Ishii J, Izawa K, Matsumura S, Wakamura K, Tanino T, et al (2009) A simple and immediate method for simultaneously evaluating expression level and plasmid maintenance in yeast. J Biochem 145: 701–708. 19237442 10.1093/jb/mvp028

[36] Ishii J, Matsumura S, Kimura S, Tatematsu K, Kuroda S, et al (2006) Quantitative and dynamic analyses of G protein-coupled receptor signaling in yeast using Fus1, enhanced green fluorescence protein (EGFP), and His3 fusion protein. Biotechnol Prog 22: 954–960. 16889369 10.1021/bp0601387

