# Supplementary material for: Rapid, Facile Detection of Heterodimer Partners for Target Human G-Protein-Coupled Receptors Using a Modified Split-Ubiquitin Membrane Yeast Two-Hybrid System
Source: PLoS One. 2013 Jun 21;8(6):e66793. doi: 10.1371/journal.pone.0066793 (PMC3689660; doi:10.1371/journal.pone.0066793)
Supplement: Table S1 — List of oligonucleotides. (PDF) [file pone.0066793.s006.pdf]

Rapid, facile detection of heterodimer partners for target human G-protein-coupled receptors using a modified split-ubiquitin membrane yeast two-hybrid system

Table S1. List of oligonucleotides

**Yeast strain construction**		
	**Name**	**Sequence**
	dSTE20up-URA3_fw	5’-GCAAGCAACCCAAACTTCTTCCCTTCACTGCCTCACACCCCATCCTAAATATC CCACAAGATCCTCGACTAATACAAGAATTTTTTGTTCTTTTTTTTGA
	dSTE20up-URA3_rv	5’-CTTGTGGGATATTTAGGATGGGGTAATAACTGATATAATT
	dSTE20dn_fw	5’-AATTATATCAGTTATTACCCCATCCTAAATATCCCACAAGATCCTCGACTAATAC AAGAAACGTAGCAAGCAGGGTACAC
	dSTE20dn_rv	5’-CTCCTTCGTTACATAGAACGCAGACAGATG
	dSTE11up-URA3_fw	5’-TATTCATATTTACACACATGCATAAAGAGAGACCACTTAATAAAGCTAGTATGAT AAGATCACCGGTAGACGAAATATACTTTTTTGTTCTTTTTTTTGA
	dSTE11up-URA3_rv	5’-ATCTTATCATACTAGCTTTAGGGTAATAACTGATATAATT
	dSTE11dn_fw	5’-AATTATATCAGTTATTACCCTAAAGCTAGTATGATAAGATCACCGGTAGACGAA ATATACAAAAGGGCTACTTATTAATT
	dSTE11dn_rv	5’-TCACCTCGCAAAGAAAGGCCTGTTTCTTCG
	dSTE2up-URA3_fw	5’-TTTCTTTTCACCTGCTCTGGCTATAATTATAATTGGTTACTTAAAAATGCACCGT TAAGAACCATATCCAAGAATCAAAATTTTTTGTTCTTTTTTTTGA
	dSTE2up-URA3_rv	5’-TCTTAACGGTGCATTTTTAAGGGTAATAACTGATATAATT
	dSTE2dn_fw	5’-AATTATATCAGTTATTACCCTTAAAAATGCACCGTTAAGAACCATATCCAAGAAT CAAAATCAAAATTTACGGCTTTGAAAAAGTAATTTCGTGACCTTC
	dSTE2dn_rv	5’-AAGATTAACTGTATATATTGCCTGAGAGTTCTAGATCATG
**Plasmid construction**		
	**Name**	**Sequence**
o1	SacII_PHO5p_fw	5’-AAAACCGCGGTTTTCTTTGTCTGCACAAAG
o2	XbaI_PHO5p_rv	5’-AAAATCTAGATGGTAATCTCGAATTTGCTT
o3	SacII_TPI1p_fw	5’-AAAACCGCGGCTACTTATTCCCTTCGAGAT
o4	XbaI_TPI1p_rv	5’-AAAATCTAGATTTTAGTTTATGTATGTGTT
o5	SacII_TDH3p_fw	5’-GGGGCCGCGGGAATAAAAAACACGCTTTTT
o6	XbaI_TDH3p_rv	5’-CCCCTCTAGATTTGTTTGTTTATGTGTGTT
o7	XbaI_STE2_fw	5’-AAAATCTAGAATGTCTGATGCGGCTCCTTC
o8	HindIII_lin_STE2_rv	5’-GGGGAAGCTTGAACCTCCGCCACCTGATAAATTATTATTATCTTCAG
o9	SpeI_STE2_fw	5’-GGGGACTAGTATGTCTGATGCGGCTCCTTC
o10	EcoRI_lin_STE2_rv	5’-GGGGGAATTCGGAACCTCCGCCACCTGATAAATTATTATTATCTTCAG
o11	HindIII_lin_STE2-304_rv	5’-GGGGAAGCTTGAACCTCCGCCACCTGATTTGGATGCATTATTAGCAG
o12	EcoRI_lin_STE2-304_rv	5’-GGGGGAATTCGGAACCTCCGCCACCTGATTTGGATGCATTATTAGCAG
o13	HindIII_lin_STE2-236_rv	5’-GGGGAAGCTTGAACCTCCGCCACCTGAAAGGAATCTTCTTGATCTAA
o14	EcoRI_lin_STE2-236_rv	5’-GGGGGAATTCGGAACCTCCGCCACCTGAAAGGAATCTTCTTGATCTAA
o15	XbaI_STE2-237_fw	5’-GGGGTCTAGAATGGGTCTCAAGCAGTTCGA
o16	SpeI_STE2-237_fw	5’-GGGGACTAGTATGGGTCTCAAGCAGTTCGA
o17	SpeI_HXT1sm_fw	5’-GGGGACTAGTATGAACTCAACTCCCGATCTAATATCTCCT
o18	EcoRI_lin_HXT1_rv	5’-GGGGGAATTCGGAACCTCCGCCACCTGATTTCCTGCTAAACAAACTCTTG
o19	XbaI_GABBR1a_fw	5’-AAAATCTAGAATGTTGCTGCTGCTGCTACT
o20	HindIII_lin_GABBR1a_rv	5’-GGGGAAGCTTGAACCTCCGCCACCTGACTTATAAAGCAAATGCACTC

o21	SpeI_GABBR1a_fw	5’-AAAAACTAGTATGTTGCTGCTGCTGCTACT
o22	EcoRI_lin_GABBR1a_rv	5’-GGGGGAATTCGGAACCTCCGCCACCTGACTTATAAAGCAAATGCACTC
o23	XbaI_GABBR2_fw	5’-AAAATCTAGAATGGCTTCCCCGCGGAGCTC
o24	HindIII_lin_GABBR2_rv	5’-TTTTAAGCTTGAACCTCCGCCACCTGACAGGCCCGAGACCATGACTC
o25	SpeI_GABBR2_fw	5’-AAAAACTAGTATGGCTTCCCCGCGGAGCTC
o26	EcoRI_lin_GABBR2_rv	5’-TTTTGAATTCGGAACCTCCGCCACCTGACAGGCCCGAGACCATGACTC
o27	XbaI_AGTR1_fw	5’-AAAATCTAGAATGATTCTCAACTCTTCTAC
o28	HindIII_lin_AGTR1_rv	5’-GGGGAAGCTTGAACCTCCGCCACCTGACTCAACCTCAAAACATGGTG
o29	SpeI_AGTR1_fw	5’-GGGGACTAGTATGATTCTCAACTCTTCTAC
o30	EcoRI_lin_AGTR1_rv	5’-GGGGGAATTCGGAACCTCCGCCACCTGACTCAACCTCAAAACATGGTG
o31	SpeI_AGTR2_fw	5’-TTTTACTAGTATGAAGGGCAACTCCACCCT
o32	EcoRI_lin_AGTR2_rv	5’-TTTTGAATTCGGAACCTCCGCCACCTGAAGACACAAAGGTCTCCATTT
o33	XbaI_MTNR1A_fw	5’-TTTTTCTAGAATGCAGGGCAACGGCAGCGC
o34	HindIII_lin_MTNR1A_rv	5’-GGGGAAGCTTGAACCTCCGCCACCTGAAACGGAGTCCACCTTTACTA
o35	SpeI_MTNR1A_fw	5’-TTTTACTAGTATGCAGGGCAACGGCAGCGC
o36	EcoRI_lin_MTNR1A_rv	5’-GGGGGAATTCGGAACCTCCGCCACCTGAAACGGAGTCCACCTTTACTA
o37	SpeI_MTNR1B_fw	5’-GGGGACTAGTATGTCAGAGAACGGCTCCTT
o38	EcoRI_lin_MTNR1B_rv	5’-AAAAGAATTCGGAACCTCCGCCACCTGAGAGAGCATCTGCCTGGTGCT
o39	XbaI_SSTR2_fw	5’-AAAATCTAGAATGGACATGGCGGATGAGCC
o40	HindIII_lin_SSTR2_rv	5’-AAAAAAGCTTGAACCTCCGCCACCTGAGATACTGGTTTGGAGGTCTC
o41	SpeI_SSTR2_fw	5’-CCCCACTAGTATGGACATGGCGGATGAGCC
o42	EcoRI_lin_SSTR2_rv	5’-AAAAGAATTCGGAACCTCCGCCACCTGAGATACTGGTTTGGAGGTCTC
o43	XbaI_SSTR5_fw	5’-AAAATCTAGAATGGAGCCCCTGTTCCCAGC
o44	HindIII_lin_SSTR5_rv	5’-TTTTAAGCTTGAACCTCCGCCACCTGACAGCTTGCTGGTCTGCATAA
o45	SpeI_SSTR5_fw	5’-AAAAACTAGTATGGAGCCCCTGTTCCCAGC
o46	EcoRI_lin_SSTR5_rv	5’-AAAAGAATTCGGAACCTCCGCCACCTGACAGCTTGCTGGTCTGCATAA
o47	XbaI_ADRB2_fw	5’-TTTTTCTAGAATGGGGCAACCCGGGAACGG
o48	HindIII_lin_ADRB2_rv	5’-TTTTAAGCTTGAACCTCCGCCACCTGACAGCAGTGAGTCATTTGTAC
o49	SpeI_ADRB2_fw	5’-AAAAACTAGTATGGGGCAACCCGGGAACGG
o50	EcoRI_lin_ADRB2_rv	5’-AAAAGAATTCGGAACCTCCGCCACCTGACAGCAGTGAGTCATTTGTAC
o51	XbaI_HTR1A_fw	5’-AAAATCTAGAATGGATGTGCTCAGCCCTGG
o52	HindIII_lin_HTR1A_rv	5’-TTTTAAGCTTGAACCTCCGCCACCTGACTGGCGGCAGAACTTACACT
o53	SpeI_HTR1A_fw	5’-GGGGACTAGTATGGATGTGCTCAGCCCTGG
o54	EcoRI_lin_HTR1A_rv	5’-GGGGGAATTCGGAACCTCCGCCACCTGACTGGCGGCAGAACTTACACT
o55	SpeI_EDNRB_fw	5’-GGGGACTAGTATGCAGCCGCCTCCAAGTCT
o56	ClaI_lin_EDNRB_rv	5’-GGGGATCGATGGAACCTCCGCCACCTGAAGATGAGCTGTATTTATTAC
o57	SpeI_NTSR1_fw	5’-GGGGACTAGTATGCGCCTCAACAGCTCCGC
o58	EcoRI_lin_NTSR1_rv	5’-AAAAGAATTCGGAACCTCCGCCACCTGAGTACAGCGTCTCGCGGGTGG
o59	SpeI_NTSR2_fw	5’-TTTTACTAGTATGGAAACCAGCAGCCCGCG
o60	EcoRI_lin_NTSR2_rv	5’-AAAAGAATTCGGAACCTCCGCCACCTGAGGTCCGGGTTTCTGGGGGAT
