# Supplementary material for: Rapid, Facile Detection of Heterodimer Partners for Target Human G-Protein-Coupled Receptors Using a Modified Split-Ubiquitin Membrane Yeast Two-Hybrid System
Source: PLoS One. 2013 Jun 21;8(6):e66793. doi: 10.1371/journal.pone.0066793 (PMC3689660; doi:10.1371/journal.pone.0066793)
Supplement: Table S2 — List of plasmids. (PDF) [file pone.0066793.s007.pdf]

Rapid, facile detection of heterodimer partners for target human G-protein-coupled receptors using a modified split-ubiquitin membrane yeast two-hybrid system

Table S2. List of plasmids

Plasmid name	Expressed protein[a,b]	Vector backbone	Promoter	Source
**Bait**				
pCCW-Alg5	Alg5-Cub	–	CYC1	Dualsystems Biotech AG
pBT3-C	Cub	–	CYC1	Dualsystems Biotech AG
pBPH3-C	Cub	pBT3-C	PHO5	This study
pBTP3-C	Cub	pBT3-C	TPI1	This study
pBTD3-C	Cub	pBT3-C	TDH3	This study
pBT3-STE2	Ste2(full length)-Cub	pBT3-C	CYC1	This study
pBPH3-STE2	Ste2(full length)-Cub	pBPH3-C	PHO5	This study
pBTP3-STE2	Ste2(full length)-Cub	pBTP3-C	TPI1	This study
pBTD3-STE2	Ste2(full length)-Cub	pBTD3-C	TDH3	This study
pBT3-STE2ΔC	Ste2(aa 1-304)-Cub	pBT3-C	CYC1	This study
pBTP3-STE2ΔC	Ste2(aa 1-304)-Cub	pBTP3-C	TPI1	This study
pBT3-STE2TM1-5	Ste2(aa 1-236)-Cub	pBT3-C	CYC1	This study
pBT3-STE2TM6-7	Ste2(aa 237-304)-Cub	pBT3-C	CYC1	This study
pBTP3-GABBR1a	GABBR1a-Cub	pBTP3-C	TPI1	This study
pBTP3-GABBR2	GABBR2-Cub	pBTP3-C	TPI1	This study
pBTD3-GABBR2	GABBR2-Cub	pBTD3-C	TDH3	This study
pBT3-AGTR1	AGTR1-Cub	pBT3-C	CYC1	This study
pBTP3-AGTR1	AGTR1-Cub	pBTP3-C	TPI1	This study
pBT3-MTNR1A	MTNR1A-Cub	pBT3-C	CYC1	This study
pBPH3-MTNR1A	MTNR1A-Cub	pBPH3-C	PHO5	This study
pBT3-SSTR2	SSTR2-Cub	pBT3-C	CYC1	This study
pBPH3-SSTR2	SSTR2-Cub	pBPH3-C	PHO5	This study
pBTP3-SSTR2	SSTR2-Cub	pBTP3-C	TPI1	This study
pBTD3-SSTR2	SSTR2-Cub	pBTD3-C	TDH3	This study
pBTD3-SSTR5	SSTR5-Cub	pBTD3-C	TDH3	This study
pBT3-ADRB2	ADRB2-Cub	pBT3-C	CYC1	This study
pBTP3-ADRB2	ADRB2-Cub	pBTP3-C	TPI1	This study
pBTD3-ADRB2	ADRB2-Cub	pBTD3-C	TDH3	This study
pBPH3-HTR1A	HTR1A-Cub	pBPH3-C	PHO5	This study
pBTP3-HTR1A	HTR1A-Cub	pBTP3-C	TPI1	This study
**Prey**				
pAI-Alg5	Alg5-Nub*I*	–	ADH1	Dualsystems Biotech AG
pPR3-C	Nub*G*	–	ADH1	Dualsystems Biotech AG
pPR3-STE2	Ste2(full length)-Nub*G*	pPR3-C	ADH1	This study
pPR3-STE2ΔC	Ste2(aa 1-304)-Nub*G*	pPR3-C	ADH1	This study
pPR3-STE2TM1-5	Ste2(aa 1-236)-Nub*G*	pPR3-C	ADH1	This study
pPR3-STE2TM6-7	Ste2(aa 237-304)-Nub*G*	pPR3-C	ADH1	This study
pPR3-HXT1	Hxt1(full length)-Nub*G*	pPR3-C	ADH1	This study
pPR3-GABBR1a	GABBR1a-Nub*G*	pPR3-C	ADH1	This study
pPR3-GABBR2	GABBR2-Nub*G*	pPR3-C	ADH1	This study

pPR3-AGTR1	AGTR1-Nub*G*	pPR3-C	ADH1	This study
pPR3-AGTR2	AGTR2-Nub*G*	pPR3-C	ADH1	This study
pPR3-MTNR1A	MTNR1A-Nub*G*	pPR3-C	ADH1	This study
pPR3-MTNR1B	MTNR1B-Nub*G*	pPR3-C	ADH1	This study
pPR3-SSTR2	SSTR2-Nub*G*	pPR3-C	ADH1	This study
pPR3-SSTR5	SSTR5-Nub*G*	pPR3-C	ADH1	This study
pPR3-ADRB2	ADRB2-Nub*G*	pPR3-C	ADH1	This study
pPR3-HTR1A	HTR1A-Nub*G*	pPR3-C	ADH1	This study
pPR3-EDNRB	EDNRB-Nub*G*	pPR3-C	ADH1	This study
pPR3-NTSR1	NTSR1-Nub*G*	pPR3-C	ADH1	This study
pPR3-NTSR2	NTSR2-Nub*G*	pPR3-C	ADH1	This study

a) Cub indicates the Cub-LexA-VP16 fusion protein

b) Nub*G* indicates Nub with I13G mutation
