# Supplementary material for: Rapid, Facile Detection of Heterodimer Partners for Target Human G-Protein-Coupled Receptors Using a Modified Split-Ubiquitin Membrane Yeast Two-Hybrid System
Source: PLoS One. 2013 Jun 21;8(6):e66793. doi: 10.1371/journal.pone.0066793 (PMC3689660; doi:10.1371/journal.pone.0066793)
Supplement: Table S3 — List of prey GPCR library. (PDF) [file pone.0066793.s008.pdf]

PLoS ONE
Supporting Information:

Rapid, facile detection of heterodimer partners for target human G-protein-coupled receptors using a modified split-ubiquitin membrane yeast two-hybrid system

Table S3. List of prey GPCR library

Prey plasmid name
pPR3-AGTR1
pPR3-ADRB2
pPR3-HTR1A
pPR3-SSTR2
pPR3-SSTR5
pPR3-EDNRB
pPR3-NTSR1
pPR3-NTSR2
pPR3-C(Control)
