# Supplementary material for: Rapid, Facile Detection of Heterodimer Partners for Target Human G-Protein-Coupled Receptors Using a Modified Split-Ubiquitin Membrane Yeast Two-Hybrid System
Source: PLoS One. 2013 Jun 21;8(6):e66793. doi: 10.1371/journal.pone.0066793 (PMC3689660; doi:10.1371/journal.pone.0066793)
Supplement: Document S1 — Supplementary Materials and Methods (Plasmid constructions for supporting information). (PDF) [file pone.0066793.s009.pdf]

Rapid, facile detection of heterodimer partners for target human G-protein-coupled receptors using a modified split-ubiquitin membrane yeast two-hybrid system

Document S1. Supplementary Materials and Methods (Plasmid constructions for supporting information)

All plasmids used in this study are summarized in Table S2. All oligonucleotides are listed in Table S1. The bait proteins (X) were fused with a C-terminal ubiquitin moiety linked to an artificial transcription factor (X-Cub) in pBT3-C (Dualsystems Biotech AG, Schlieren, Switzerland). The prey proteins (Y) were fused with an N-terminal moiety of split-ubiquitin with I13G mutation (Y-Nub*G*) in pPR3-C (Dualsystems Biotech AG).

Bait vectors: For bait vectors, several promoters exhibiting distinctive expression strength were substituted for the original weak *CYC1* promoter of pBT3-C as follows.
PHO5 promoter (stronger than *CYC1* promoter) was PCR-amplified with oligonucleotides 01 and 02. The *Sac*II-*Xba*I *PHO5* promoter was inserted at the *Sac*II-*Xba*I site on pBT3-C, resulting in the plasmid pBPH3-C.
TPI1 promoter (stronger than *PHO5* promoter) was PCR-amplified with oligonucleotides 03 and 04. The *Sac*II-*Xba*I *TPI1* promoter was inserted at the *Sac*II-*Xba*I site on pBT3-C, resulting in the plasmid pBTP3-C.
TDH3 promoter (stronger than *TPI1* promoter) was PCR-amplified with oligonucleotides 05 and 06. The *Sac*II-*Xba*I *TDH3* promoter was inserted at the *Sac*II-*Xba*I site on pBT3-C, resulting in the plasmid pBTD3-C.

GPCR expression plasmids: The bait and prey plasmids used for the expression of GPCRs were constructed as follows. Full length *STE2* genes encoding yeast pheromone receptor were PCR-amplified with oligonucleotide pairs: 07 and 08; 09 and 010. The *Xba*I-*Hind*III *STE2* gene fragments were inserted at the *Xba*I-*Hind*III site on pBT3-C, pBPH3-C, pBTP3-C and pBTD3-C, resulting in the plasmids pBT3-STE2, pBPH3-STE2, pBTP3-STE2 and pBTD3-STE2, respectively. The *Spe*I-*Eco*RI *STE2* gene fragment was inserted at the *Spe*I-*Eco*RI site on pPR3-C, resulting in the plasmid pPR3-STE2.
Truncated *STE2* genes that lack the C-terminal domain (Ste2ΔC) were PCR-amplified

with oligonucleotide pairs: o7 and o11; o9 and o12. The *Xba*I-*Hind*III *STE2ΔC* gene fragments were inserted at the *Xba*I-*Hind*III site on pBT3-C and pBTP3-C, resulting in the plasmid pBT3-STE2ΔC and pBTP3-STE2ΔC, respectively. The *Spe*I-*Eco*RI *STE2ΔC* gene fragment was inserted at the *Spe*I-*Eco*RI site on pPR3-C, resulting in the plasmid pPR3-STE2ΔC.

Deletional *STE2* genes that lack the domains from TM6 until C-terminal tail (TM1-5) were PCR-amplified with oligonucleotide pairs: o7 and o13; o9 and o14. The *Xba*I-*Hind*III *TM1-5* gene fragment was inserted at the *Xba*I-*Hind*III site on pBT3-C, resulting in the plasmid pBT3-STE2TM1-5. The *Spe*I-*Eco*RI *TM1-5* gene fragment was inserted at the *Spe*I-*Eco*RI site on pPR3-C, resulting in the plasmid pPR3-STE2TM1-5.

Deletional *STE2* genes that lack the domains from the N-terminal tail to TM5 and C-terminal domain (TM6-7) were PCR-amplified with oligonucleotide pairs: o15 and o11; o16 and o12. The *Xba*I-*Hind*III *TM6-7* gene fragment was inserted at the *Xba*I-*Hind*III site on pBT3-C, resulting in the plasmid pBT3-STE2TM6-7. The *Spe*I-*Eco*RI *TM6-7* gene fragment was inserted at the *Spe*I-*Eco*RI site on pPR3-C, resulting in the plasmid pPR3-STE2TM6-7.

The *HXT1* gene encoding glucose transporter was PCR-amplified with oligonucleotides o17 and o18. The *Spe*I-*Eco*RI *HXT1* gene fragment was inserted at the *Spe*I-*Eco*RI site on pPR3-C, resulting in the plasmid pPR3-HXT1.

GABBR1a genes encoding $GABA_{B1a}$ receptor were PCR-amplified with oligonucleotide pairs: o19 and o20; o21 and o22. The *Xba*I-*Hind*III *GABBR1a* gene fragment was inserted at the *Xba*I-*Hind*III site on pBTP3-C, resulting in the plasmid pBTP3-GABBR1a. The *Spe*I-*Eco*RI *GABBR1a* gene fragment was inserted at the *Spe*I-*Eco*RI site on pPR3-C, resulting in the plasmid pPR3-GABBR1a.

GABBR2 genes encoding $GABA_{B2}$ receptor were PCR-amplified with oligonucleotide pairs: o23 and o24; o25 and o26. The *Xba*I-*Hind*III *GABBR2* gene fragments were inserted at the *Xba*I-*Hind*III site on pBTP3-C and pBTD3-C, resulting in the plasmid pBTP3-GABBR2 and pBTD3-GABBR2, respectively. The *Spe*I-*Eco*RI *GABBR2* gene fragment was inserted at the *Spe*I-*Eco*RI site on pPR3-C, resulting in the plasmid pPR3-GABBR2.

AGTR1 genes encoding AT_1 (angiotensin type 1) receptor were PCR-amplified with oligonucleotide pairs: o27 and o28; o29 and o30. The *Xba*I-*Hind*III *AGTR1* gene fragments were inserted at the *Xba*I-*Hind*III site on pBT3-C and pBTP3-C, resulting in the plasmid pBT3-AGTR1 and pBTP3-AGTR1, respectively. The *Spe*I-*Eco*RI *AGTR1* gene fragment was inserted at the *Spe*I-*Eco*RI site on pPR3-C, resulting in the plasmid pPR3-AGTR1.

AGTR2 gene encoding AT_2 (angiotensin type 2) receptor was PCR-amplified with oligonucleotides o31 and o32. The *Spe*I-*Eco*RI *AGTR2* gene fragment was inserted at the *Spe*I-*Eco*RI site on pPR3-C, resulting in the plasmid pPR3-AGTR2.

MTNR1A genes encoding MT_1 (melatonin 1A) receptor were PCR-amplified with oligonucleotide pairs: o33 and o34; o35 and o36. The *Xba*I-*Hind*III *MTNR1A* gene fragments were inserted at the *Xba*I-*Hind*III site on pBT3-C, resulting in the plasmid pBT3-MTNR1A and pBPH3-MTNR1A, respectively. The *Spe*I-*Eco*RI *MTNR1A* gene fragment was inserted at the *Spe*I-*Eco*RI site on pPR3-C, resulting in the plasmid pPR3-MTNR1A.

MTNR1B gene encoding MT_2 (melatonin 1B) receptor was PCR-amplified with oligonucleotides o37 and o38. The *Spe*I-*Eco*RI *MTNR1B* gene fragment was inserted at the *Spe*I-*Eco*RI site on pPR3-C, resulting in the plasmid pPR3-MTNR1B.

SSTR2 genes encoding somatostatin receptor 2 were PCR-amplified with oligonucleotide pairs: o39 and o40; o41 and o42. The *Xba*I-*Hind*III *SSTR2* gene fragments were inserted at the *Xba*I-*Hind*III site on pBT3-C, pBPH3-C, pBTP3-C and pBTD3-C, resulting in the plasmid pBT3-SSTR2, pBPH3-SSTR2, pBTP3-SSTR2 and pBTD3-SSTR2, respectively. The *Spe*I-*Eco*RI *SSTR2* gene fragment was inserted at the *Spe*I-*Eco*RI site on pPR3-C, resulting in the plasmid pPR3-SSTR2.

SSTR5 genes encoding somatostatin receptor 5 were PCR-amplified with oligonucleotide pairs: o43 and o44; o45 and o46. The *Xba*I-*Hind*III *SSTR5* gene fragment was inserted at the *Xba*I-*Hind*III site on pBTD3-C, resulting in the plasmid pBTD3-SSTR5. The *Spe*I-*Eco*RI *SSTR5* gene fragment was inserted at the *Spe*I-*Eco*RI site on pPR3-C, resulting in the plasmid pPR3-SSTR5.

ADRB2 genes encoding β_2-adrenergic receptor were PCR-amplified with

oligonucleotide pairs: 047 and 048; 049 and 050. The *Xba*I-*Hind*III *ADRB2* gene fragments were inserted at the *Xba*I-*Hind*III site on pBT3-C, pBTP3-C and pBTD3-C, resulting in the plasmid pBT3-ADRB2, pBTP3-ADRB2 and pBTD3-ADRB2, respectively. The *Spe*I-*Eco*RI *ADRB2* gene fragment was inserted at the *Spe*I-*Eco*RI site on pPR3-C, resulting in the plasmid pPR3-ADRB2.

HTR1A genes encoding 5-hydroxytryptamine (serotonin) receptor 1A were PCR-amplified with oligonucleotide pairs: 051 and 052; 053 and 054. The *Xba*I-*Hind*III *HTR1A* gene fragments were inserted at the *Xba*I-*Hind*III site on pBPH3-C and pBTP3-C, resulting in the plasmid pBPH3-HTR1A and pBTP3-HTR1A, respectively. The *Spe*I-*Eco*RI *HTR1A* gene fragment was inserted at the *Spe*I-*Eco*RI site on pPR3-C, resulting in the plasmid pPR3-HTR1A.

EDNRB gene encoding endothelin receptor type B was PCR-amplified with oligonucleotides 055 and 056. The *Spe*I-*Cla*I *EDNRB* gene fragment was inserted at the *Spe*I-*Cla*I site on pPR3-C, resulting in the plasmid pPR3-EDNRB.

NTSR1 gene encoding neurotensin receptor 1 was PCR-amplified with oligonucleotides 057 and 058. The *Spe*I-*Eco*RI *NTSR1* gene fragment was inserted at the *Spe*I-*Eco*RI site on pPR3-C, resulting in the plasmid pPR3-NTSR1.

NTSR2 gene encoding neurotensin receptor 2 was PCR-amplified with oligonucleotides 059 and 060. The *Spe*I-*Eco*RI *NTSR2* gene fragment was inserted at the *Spe*I-*Eco*RI site on pPR3-C, resulting in the plasmid pPR3-NTSR2.
